# Tsetse EP Protein Protects the Fly Midgut from Trypanosome Establishment

**DOI:** 10.1371/journal.ppat.1000793

**Published:** 2010-03-05

**Authors:** Lee R. Haines, Stella M. Lehane, Terry W. Pearson, Michael J. Lehane

**Affiliations:** 1 Liverpool School of Tropical Medicine, Liverpool, United Kingdom; 2 Department of Biochemistry and Microbiology, University of Victoria, Victoria, British Columbia, Canada; Institut Pasteur, France

## Abstract

African trypanosomes undergo a complex developmental process in their tsetse fly vector before transmission back to a vertebrate host. Typically, 90% of fly infections fail, most during initial establishment of the parasite in the fly midgut. The specific mechanism(s) underpinning this failure are unknown. We have previously shown that a *Glossina*-specific, immunoresponsive molecule, tsetse EP protein, is up regulated by the fly in response to gram-negative microbial challenge. Here we show by knockdown using RNA interference that this tsetse EP protein acts as a powerful antagonist of establishment in the fly midgut for both *Trypanosoma brucei brucei* and *T. congolense*. We demonstrate that this phenomenon exists in two species of tsetse, *Glossina morsitans morsitans* and *G. palpalis palpalis*, suggesting tsetse EP protein may be a major determinant of vector competence in all *Glossina* species. Tsetse EP protein levels also decline in response to starvation of the fly, providing a possible explanation for increased susceptibility of starved flies to trypanosome infection. As starvation is a common field event, this fact may be of considerable importance in the epidemiology of African trypanosomiasis.

## Author Summary

In Africa, tsetse flies transmit the trypanosomes causing the devastating diseases sleeping sickness in man and nagana in domesticated animals. These diseases are major causes of underdevelopment in Africa. Paradoxically, most, but not all, flies are resistant to infection with trypanosomes, but we do not have a clear picture of how flies fight off trypanosomes. Here we show that a particular, tsetse-specific immune responsive protein called tsetse EP acts as a powerful antagonist of trypanosome establishment in the fly midgut. It is known that starvation of flies leads to an increase in their susceptibility to trypanosomes and this may be a considerable factor in the epidemiology of the disease in Africa. Here we demonstrate that starvation leads to a decrease in tsetse EP levels, which may explain how starvation of the fly works to increase its susceptibility.

## Introduction

African trypanosomes are protozoan parasites that cause sleeping sickness in humans and nagana in domestic livestock in sub-Saharan Africa. An epidemic involving several hundred thousand people that spread through Sudan, the Central African Republic, DRC and Angola in the 1990's, demonstrated how socially and economically devastating these diseases are [1]. Trypanosomes kill more than 3 million cattle annually and those animals that survive display low productivity due to the wasting effects of the disease [2]. The annual losses from trypanosomiasis in cattle amount to more than US $4.5 billion [3]. Trypanosomes, by influencing food production, natural resource utilization and the pattern of human settlement, are thus seen by the African Union as one of the greatest constraints to Africa's socio-economic development [4].

African trypanosomes are cyclically transmitted by tsetse flies (*Glossina* spp.). *Trypanosoma brucei* and *T. congolense* undergo a complex cycle of development in the tsetse beginning almost immediately after ingestion of an infected bloodmeal when trypanosome bloodstream forms (BSF) differentiate to the procyclic form in the fly midgut lumen [5],[6],[7]. For the first three days following infection all flies contain trypanosomes. Between days 4 and 5 trypanosome infections are eliminated from most flies [7] through a process we term self-cure. The identified factors that influence vector competence (the ability to transmit parasites) include the age of the fly, the number of bloodmeals taken and the activation of fly immune processes, with both antimicrobial (host defense) peptides [8], and lectins [9],[10],[11] implicated in parasite-vector interactions. More recently, antioxidants have been shown to increase fly susceptibility when administered to flies in an infective bloodmeal [12]. Most mature tsetse are resistant to trypanosome infection although the mechanisms involved in elimination of trypanosomes from the fly midgut (self-cure) are not understood [13].

As *T. brucei* BSF trypanosomes transform in the tsetse midgut the trypanosome surface coat changes from variant surface glycoproteins (VSG) to procyclins. At first the procyclins are a mixture of GPEET and EP forms and then expression of GPEET becomes repressed [14]. Our attention has been drawn to a fly protein called tsetse EP (accession number CAC86027), named for the extensive glutamic acid-proline dipeptide repeats that in *Glossina morsitans morsitans* comprise more than 40% of its length. The repeat section of this molecule shows remarkable sequence identity to the repeat section of the EP form of procyclin surface coat molecules of *T. b. brucei* [14]. These repeats are very rare in the protein databases and their co-incidence in two species showing such a close biological relationship is remarkable. Our knowledge of tsetse EP is limited although we do know that it is strongly up regulated following fly challenge with Gram-negative bacteria [15] suggesting a possible function in the insect immune response. In addition up regulation of the immune response by injection of *E. coli* also leads to a significant reduction in trypanosome prevalence [8],[16]. For these reasons we have undertaken a series of experiments to see if these observations are connected. We provide evidence that tsetse EP protein has a powerful role in protecting the tsetse fly midgut from trypanosome infection.

## Results

### Where is tsetse EP protein produced within the midgut of the fly?

**Figure 1:**
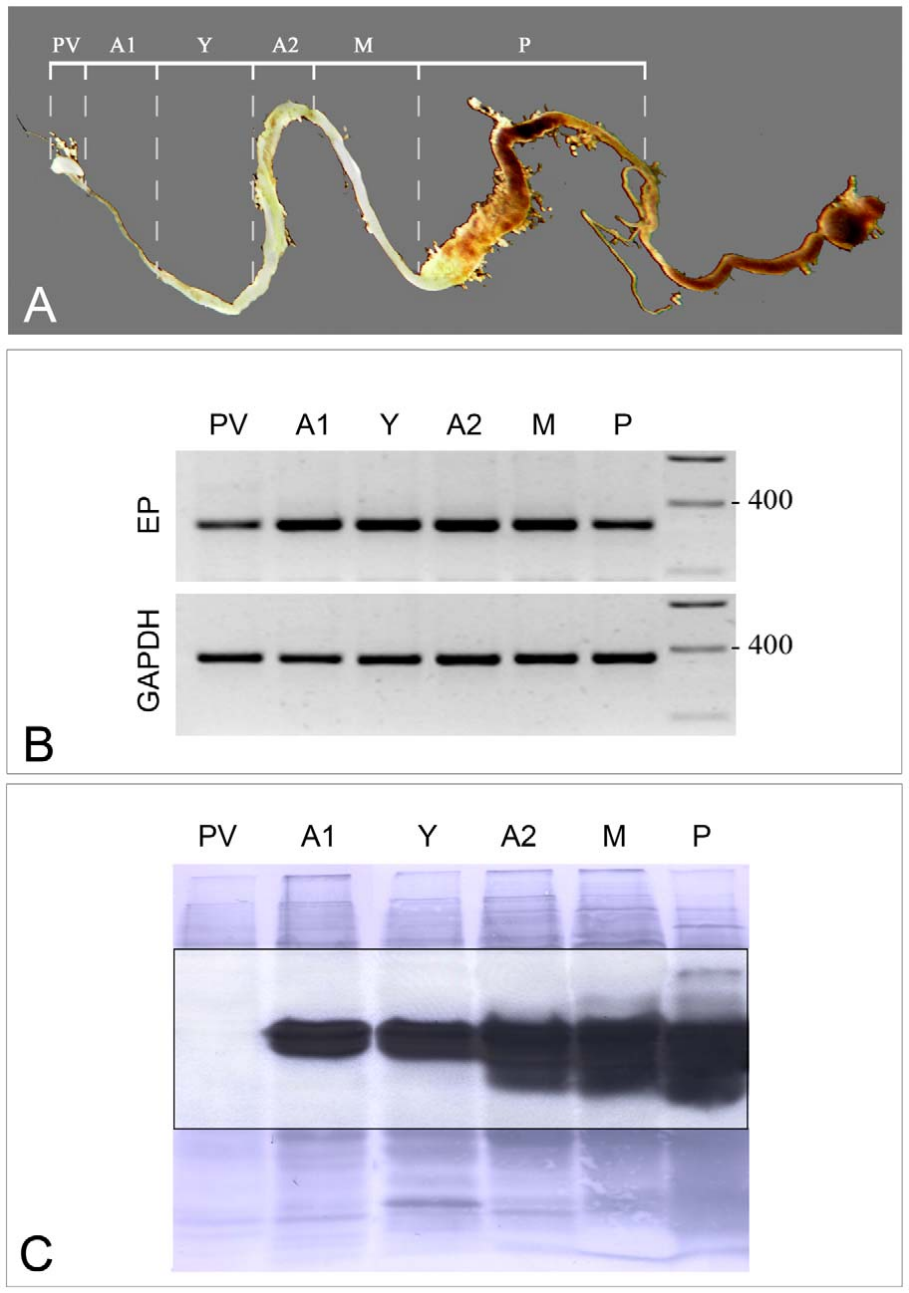
Levels of tsetse EP transcripts and proteins in sectioned midguts were determined by semi-quantitative RT-PCR and immunoblotting. (A) Photograph of tsetse midgut depicting dissection lines. (B) Transcript profiles of midgut sections probed with tsetse EP and GAPDH (control) primers. (C) Immunoblot of midgut tissues (collected from a pool of five male *G. m. morsitans*) using mAb 247 (anti-EP repeat). PV  =  proventriculus; A1  =  midgut anterior to the bacteriome; Y  =  bacteriome; A2  =  anterior midgut posterior to the bacteriome; M  =  mid-midgut; P  =  posterior midgut.

Tsetse midguts were carefully dissected into distinct structural regions (Figure 1, Panel A) to determine the location of tsetse EP mRNA and protein. Tsetse EP transcripts were detected in all sections tested. However, lower levels were consistently observed in the proventriculus (PV) (Figure 1, Panel B). The Western blot (Figure 1, Panel C) overlay of the nigrosine-stained PVDF with the autoluminogram revealed the strong presence of tsetse EP protein in all tissues except for the PV. Given the presence of tsetse EP transcript in the PV we conclude that tsetse EP protein was either not produced in PV, was rapidly turned over in that organ or was rapidly translocated from there into the anterior gut. Similarly, tsetse EP transcript was detected in salivary glands from teneral and fed flies [17] but tsetse EP protein was only weakly detected by immunoblotting suggesting it may be rapidly translocated to midgut.

### Design of dsRNA for tsetse EP knockdown

**Figure 2:**
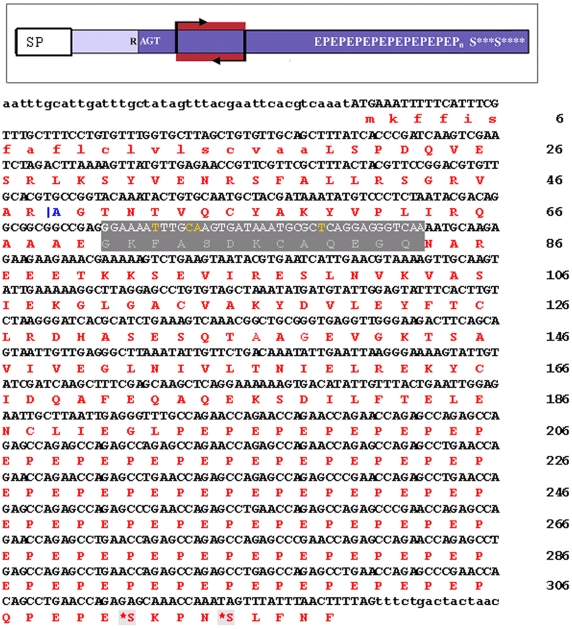
*G. m. morsitans* tsetse EP protein. The top panel is a schematic of the tsetse EP protein. SP  =  signal peptide. R AGT indicates the N-terminus (tryptic cleavage site) resulting in the mature protein. The dark blue represents the mature protein and the highlight red region (bracketed with arrows) indicates the target region for RNAi. In the sequence below, the predicted signal peptide is shown in lower case. |A indicates the N-terminus (tryptic cleavage site) resulting in the mature protein. The grey highlighted section indicates the region amplified for RNA interference. The residues in yellow indicate sequence differences in the *G. p. palpalis* sequence when compared with the sequence of *G. m. morsitans*. The monoclonal antibody, mAb 247, will recognize EPEPEP, and thus will bind along the entire length of the C-terminal rich region (E_195_–P_306_). S* indicates potential O-glycosylation sites.

Tsetse EP protein appears to be ubiquitous in *Glossina* spp. as its presence was confirmed in eight species of tsetse previously examined by Western blotting with the anti-EP repeat antibody (mAb 247) [15]. Using the available sequence analysis of the amino acid sequence of tsetse EP protein [15],[17] we designed effective double stranded RNA for knockdown experiments (Figure 2). A protein sequence comparison (87% similarity) between two species (*G. m. morsitans* and *G. p. palpalis*) revealed that the outstanding sequence difference was in the length of the C-terminal EP repeat region [15]. The tsetse EP protein is probably a preproprotein containing a short (19 mer) hydrophobic, N-terminal signal sequence as predicted by SignalP 3.0 [18]. Amino acids 20–48 appear to be removed from the remaining peptide during an undefined maturation process as determined by mass spectrometry and N-terminal sequencing [17],[19]. The EP rich domain is extremely hydrophilic, and thus almost certainly is highly soluble in aqueous solvents. It is interesting that all 8 of the cysteine residues are situated up stream of the EP rich C-terminus, suggesting that this region may be highly folded. For our experiments, we designed dsRNA to target in RNA interference the homologous region 23 residues downstream from the N-terminus of the mature protein (Figure 2, red highlighted region: GKFASDKCAQEGQ). The dsRNA target varies only slightly between *G. m. morsitans* and *G. p. palpalis* (4/39 nucleotides differ and these are shown in yellow lettering in Figure 2). Consequently the same dsRNA construct was used to achieve gene knockdown in both species.

### Tsetse EP protein levels are greatly reduced following gene knockdown by RNA interference

**Figure 3:**
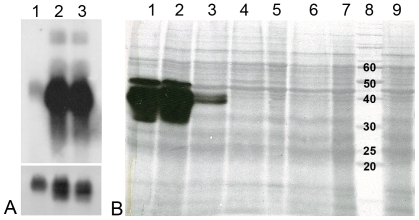
Tsetse EP mRNA and protein levels following injection of tsetse with ds*tsetseEP*. Teneral flies were injected with dsRNA, fed two bloodmeals and then starved for three days prior to dissection. Five midguts were pooled for each sample and the quantity of RNA and protein per lane was adjusted accordingly. (Panel A) Northern blot: lane 1: ds*tsetse EP* knockdown midguts; lane 2: NFW injected midguts; lane 3: control midgut tissue from non-injected flies. The lower section represents GAPDH mRNA loading controls. (Panel B) Immunoblot: Gel-separated midgut proteins (1/2 midgut equivalent) isolated from male *G. m. morsitans* injected with 2 µL of dsRNA were separated on a 12.5% gel and blotted with mAb 247 (anti-EP repeat). Lane 1: NFW control, lane 2: 8 µg ds*Amp* control, lane 3: 2 µg ds*tsetseEP*, lane 4: 4 µg ds*tsetseEP*, lane 5: 6 µg ds*tsetseEP*, lane 6: 7 µg ds*tsetseEP*, lane 7: 8 µg ds*tsetseEP*, lane 8: 10 kDa molecular mass ladder, lane 9: 10 µg ds*tsetseEP*.

During RNAi experiments mRNA levels are often extrapolated to predict protein expression levels. However, this is often misleading as the correlation between transcript abundance and protein expression levels can often vary as much as 30 fold or more, leading to a grossly distorted analysis of a biological system [20] and this may be especially true in the midgut of blood sucking insects where post-transcriptional regulation may be a common phenomenon [21]. Consequently, we measured tsetse EP levels at both the mRNA and protein levels. We show that injection of dsRNA leads to significant reductions in transcript levels compared to controls (Figure 3A). In addition, immunoblot analysis using the anti-EP repeat monoclonal antibody (mAb247) to detect the tsetse EP protein in midguts of knockdown flies showed complete elimination of the endogenous protein following a single injection of 4 or more µg of dsRNA (Figure 3B).

### Parasite infection is increased by RNAi-mediated knockdown of tsetse EP protein

We employed a reverse genetics approach to determine if tsetse EP influences parasite establishment in the midgut of the fly. We injected double-stranded RNA (dsRNA) into the thoracic haemocoel of male flies of different ages. Typically the flies were allowed to recover for 36–48 h after injecting dsRNA. This provides enough time for the dsRNA to start silencing tsetse EP protein transcription and for endogenous protein levels to decline [22]. After this point, flies were offered an infective bloodmeal containing virulent strains of either *T. b. brucei* (TSW196) or *T. congolense* (1/148) BSF. Seven days after the infectious meal the midguts were dissected, examined microscopically, snap frozen, and the number of infections was recorded (Table 1). A complicating feature of this insect system is a natural decrease in susceptibility in older flies termed the teneral phenomenon. Typically more than 50% of flies establish midgut infections when fed trypanosomes in the first bloodmeal. However, if infected in the second bloodmeal, this susceptibility declines to ∼30% of the population. By the third bloodmeal, tsetse populations are predominantly refractory to infection with typical midgut establishment rates of 10% or less (Table 1). So, we investigated flies with differing feeding histories. Tsetse EP knockdown flies, infected at all feeding time points investigated, showed statistically significant increases in susceptibility to *T. b. brucei* establishment in the midgut when compared to the controls (Table 1).

**Table 1:** Mean (± S.E.) prevalence of midgut infections in male *G. m. morsitans* (*Gmm*) or *G. p. palpalis* (*Gpp*) after RNAi knockdown using ds*tsetse EP*.

Infective Bloodmeal	No. Reps	ds*tsetse EP* () = number of flies	ds*Amp* () = number of flies	NFW* or ds*eGFP* () = number of flies	Chi-squared	p value
*Gmm* 1^st^	3	72.0±7.4 (100)	54.3±7.2 (108)	-	4.68	0.042
*Gmm* 3^rd^	3	15.1±5.6 (180)	5.6±2.3 (113)	-	5.94	0.020
*Gmm* 4^th^	3	32.4±0.6 (62)	12.0±2.3 (60)	-	5.38	0.028
*Gmm* 5^th^	3	26.0±1.0 (87)	3.7±1.7 (97)	-	18.24	<0.001
*Gmm* 10^th^	1	18.8 (32)	2.9 (35)	-	-	-
*Gpp* 1^st^	4	55.1±0.4 (125)	30.3±9.0 (99)	39.4±9.5 (99)*	16.02	<0.001
*Gpp* 5^th^	1	11.1 (36)	0 (31)	-	-	-
*Gmm* 5^th^	3	*29.7±7.2 (120)*	*1.6±1.6 (110)*	*0.9±0.9 (97)*	*52.65*	*<0.001*
*Gmm* 10^th^	1	*8.2 (49)*	*3.9 (26)*	*2.3 (44)*	*-*	*-*

Controls were either ds*Ampicillin*, ds*eGFP* or nuclease free water*. Flies were infected with either *T. b. brucei* TSW196 or *T. congolense* 1/148 (italics) blood stream forms in the indicated bloodmeal.

To determine if this phenomenon was present in other tsetse species we also investigated *G. p. palpalis*. Table 1 shows there are statistically significant increases in *T. b. brucei* establishment in the midgut of tsetse EP knockdown *G. p. palpalis*. We also conducted experiments to determine if the phenomenon extending to other trypanosome species. *T. congolense* also establishes higher midgut infections in EP knockdown flies (Table 1). Based on our current and previous [15] observations, the increase of vector competence to midgut inhabiting trypanosomes in tsetse EP knockdown flies is possibly a genus-wide phenomenon in *Glossina*.

### Tsetse EP protein levels decrease with fly starvation

**Figure 4:**
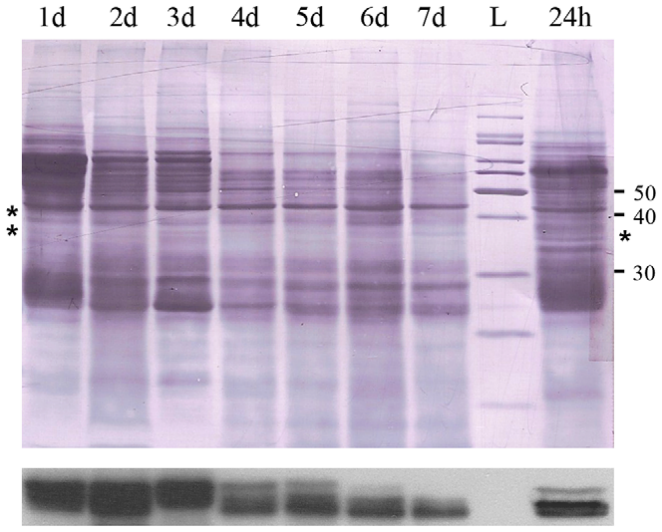
Immunoblot film lies below the nigrosine-stained PVDF. There is a decline in tsetse EP protein levels in midguts over a 7 day starvation period following the 5^th^ bloodmeal. 24 h  =  Flies starved for 7 days, fed a blood meal and then sacrificed 24 hours later. L  =  molecular mass ladder. Midgut proteins (1/2 midgut equivalent from pool of 5) were blotted with mAb 247.

Male tsetse received 5 bloodmeals prior to starvation. Flies were killed at 24 h time points, up to 7 days after the last blood meal, and individual midguts were assayed by immunoblotting using an anti-EP antibody (mAb247) (Figure 4). After 3 days of starvation a clear decline in tsetse EP protein levels is evident (Figure 4, asterisks). Fat body atrophy was also apparent in these flies when viewed with a dissection microscope. Tsetse EP protein levels increase again in flies 24 hours after feeding following a previous starvation period of 7 days (Figure 4, lane 8). We have no data to show if the starvation-induced decrease in tsetse EP protein is specific or part of a general lowering of protein levels in the midgut in response to starvation.

## Discussion

Although RNA interference is an exquisite genetic technique to knockdown target genes, the success in achieving this post-transcriptional silencing appears to be gene-specific with variability due, in part, to the half-life of endogenous target protein and unexpected lethal secondary effects from depletion of gene specific product [23]. Our unpublished observations in *Glossina* reveal that, for some genes, transcript knockdown cannot be achieved regardless of the construct designed. This may relate to the lack of a spreading mechanism in Diptera and the difficulty of dsRNA reaching cells in complex organs [22]. We have previously shown and confirm here that thoracic injections of microgram amounts of specific dsRNA can effectively depress tsetse EP transcription in the tsetse midgut for up to 2 weeks [22]. Thus, the effect of persistent tsetse EP knockdown on trypanosome midgut establishment (7–10 day experiment) could be confidently measured by microscopic examination. The data we present here shows that a tsetse molecule, tsetse EP protein, plays a role in protecting the midgut from infection with trypanosomes.

Computer analysis of the translated protein sequences from both *G. m. morsitans* (CAC86027) and *G. p. palpalis* (AAL82540), using multiple alignment tools and protein prediction algorithms, revealed that these proteins are highly conserved [15]. Including its signal peptide, tsetse EP protein from *G. m. morsitans* has a mass of 35.7 kDa and appears to form dimers and trimers and potentially larger oligomeric aggregates within the fly [15],[17]. Apart from the EP sequence the tsetse EP protein has no currently defined protein domains [17]. However a possible clue to function may be suggested by the preliminary observation of weak agglutinating activity of the large molecular complex towards freshly collected, washed rabbit red blood cells, suggesting tsetse EP putatively has some lectin activity [17]. In addition it has been demonstrated that tsetse EP protein is strongly up regulated following immune stimulation with *E. coli* [15] providing good evidence that it is part of the immune response system. Given this it is interesting to note that the Imd immune regulatory pathway mainly responds to gram negative organisms [24] and the Imd pathway has been implicated in the response of dipterans to parasite infections [16],[25].

Although all species of tsetse studied to date express tsetse EP protein [15], orthologues are not found in the *Anopheles, Aedes, Apis* or *Drosophila* genomes. A search of non-redundant databases revealed only two eukaryotic protein hits (apart from the procyclins): gi|94390895 [*Mus musculus*] and gi|109464874 [*Rattus norvegicus*]. These hypothetical proteins contain significant continuous EP repeat regions: *e.g.* 115 dipeptide repeats, representing 75% coverage of the rat protein. Unfortunately, no further functional information is available for these proteins. Remarkably, extensive regions of EP repeats (also varying in length) are contained in several procyclins that form the surface coat of procyclic trypanosomes of the *T. brucei* group [14],[26],[27]. Given the scarcity of EP repeats in organisms the chances of this happening coincidentally in trypanosomes and tsetse flies seem remote.

To examine the possibility that the tsetse EP protein and the EP procyclins from *T. b. brucei* were involved in antigenic mimicry we investigated another trypanosome species that lacks EP procyclins. The procyclic coat of *T. congolense* contains no extensive dipeptidyl EP repeats although similar anionic motifs are present [28]. Despite the absence of EP repeats, establishment of *T. congolense* is similarly affected by tsetse EP protein knockdown (Table 1). Our experiments demonstrate that tsetse EP protein can partially protect against the midgut establishment of trypanosomes from both the *Trypanozoon* and *Nannomonas* group trypanosomes and thus, strictly sequence-specific interactions in tsetse and trypanosome are not likely at play.

To assess if trypanosome establishment is altered by tsetse EP gene knockdown in tsetse species other than our *G. m. morsitans* laboratory model, we tested our RNAi protocol on *G. p. palpalis*. Knockdown of tsetse EP protein in *G. p. palpalis* also led to an increase in midgut infections (Table 1), confirming that tsetse EP protein influences trypanosome midgut establishment in both of these major vectors of trypanosomiasis. Given that tsetse EP has been demonstrated in a wide variety of *Glossina* species [15] this data suggests it may be a genus wide phenomenon.

It has been demonstrated that up regulation of the immune response by injection of *E. coli* leads to a significant reduction in the ability of trypanosomes to establish in the tsetse midgut [8],[16]. We have already demonstrated that tsetse EP protein is strongly up regulated upon introduction of Gram-negative bacteria into the fly [15]. Our demonstration here that knockdown of tsetse EP leads to increased fly susceptibility suggests that upregulation of tsetse EP protein upon injection of *E. coli* may be one explanation for the subsequent decrease in the susceptibility of the fly to trypanosomes.

It is interesting to note that older flies in field populations show unexpectedly high levels of susceptibility compared to laboratory reared flies where susceptibility rapidly declines following eclosion [29],[30] (Table 1); the reasons remain unexplained. We have demonstrated here that starvation reduces tsetse EP levels in flies (Figure 4). It has already been demonstrated that starvation of mature flies results in an increase in parasite survival in the midgut [31],[32],[33]. Consequently, starvation, which is likely to be a common phenomenon in the field, could explain the differences in susceptibility seen between field and laboratory populations of flies. The observed reduction of tsetse EP protein expression and loss of parasite resistance upon starvation may have considerable epidemiological significance in African trypanosomiasis.

In summary, this paper provides direct evidence for a tsetse-specific midgut molecule (tsetse EP), which is an antagonist of trypanosome survival in the vector. RNAi-induced knockdown of the midgut-associated, immunoresponsive tsetse EP protein increased the frequency of trypanosome establishment in the fly midgut up to more than six fold. The precise mechanism by which tsetse EP protein influences the refractorial capacity of the midgut remains to be elucidated.

## Materials and Methods

### Flies and trypanosomes

Tsetse (*G. m. morsitans*) were maintained in laboratory colony at the Liverpool School of Tropical Medicine (LSTM) at 26°C and 65–70% relative humidity. *Glossina palpalis palpalis* were supplied as puparia from the International Atomic Energy Agency (IAEA) Entomology Laboratories, Siebersdorf, Austria. Every 48 hours, male flies were fed horse blood through silicone membranes. For infectious bloodmeals blood stream forms (BSF) of *Trypanosoma brucei brucei* TSW196 MSUS/CI/78/TSW196 [CLONE A], which is a fully fly-transmissible clone and able to undergo genetic exchange [34], and *T. congolense* 1/148 (Lister 1/148; isolated from a Zebu ox, Dongo River, Nigeria, Godfrey, 1960) were added to sterile defibrinated horse blood (TCS Biosciences Ltd., Buckingham, UK). Typically 200 µL of mouse blood (containing 4×10^6^ parasites) were diluted in 5 mL of horse blood. Flies were dissected 6 days after the infectious bloodmeal. Midguts were dissected in saline on a glass slide and infection status determined by searching 10 random fields by light microscopy (125× magnification).

### dsRNA

Double stranded RNA was transcribed using a MEGAscript High Yield T7 Transcription kit (Ambion, Huntingdon, UK). *tsetseEP* templates were available as clones from the tsetse EST program [35]. A double stranded fragment of the ampicillin resistance gene (ds*AMP*) was generated using pBluescript II SK+ as template. Template DNA was removed from the transcription reaction by DNase treatment and dsRNA was purified using MEGAclear™ columns (Ambion) and eluted in nuclease free water. Eluates were concentrated in a Christ (Osterode, Germany) 2–18 rotational vacuum concentrator to approximately 5 µg per µL. Primers were designed with the 20 base core T7 promoter sequence at the 5′ end. Primer sequences used were: AmpT7A TAATACGACTCACTATAGGGTTGCCGGGAAGCTAGAGTAAGTA; AmpT7B TAATACGACTCACTATAGGGAACGCTGGTGAAAGTAAAAGATG; EPT7A TAATACGACTCACTATAGGGTTCTGGCAAACCCTCAAT; EPT7B TAATACGACTCACTATAGGGCTACGATAAATATGTCCCTCTAAT.

Borosilicate glass capillaries (2.00 mm outside diameter) were formed into a fine point using a needle puller (PC10; Narishige, Japan). To generate tsetse EP knockdowns, male flies were anaesthetized by chilling and intrathoracically injected with 10 µg (2 µL volume) of dsRNA buffered in nuclease-free water.

### RT-PCR

The primers used in semi-quantitative RT-PCR reactions for determination of transcript abundance in tsetse tissues were: *Gm GAPDHA* CTCAGCTTCTGTGCGTTG (Tm°C 67); *Gm GAPDHB* AGAGTGCCACCTACGATG (Tm°C 67); *GmmEPA* ACCGTTCGTTCGCTTTACTAC (Tm°C 47); *GmmEPB* ACCCGCAGCCGTTTGACTTTC (Tm°C 51).

Total RNA was extracted from individual tissues using Trizol (Invitrogen, Paisley UK) and treated with RQ1 RNase-Free DNase. RNA was quantified using a Nanodrop ND-1000 (Wilmington, DE) spectrophotometer. A Promega Access RT-PCR System (Promega, Southampton, UK) was used for amplification of transcripts. *G. m. morsitans* GAPDH (Accession number DQ016434) was used to normalize samples. PCR cycling conditions were: 48°C for 45 minutes, 94°C for 2 minutes, followed by 30 cycles of 94°C for 30 seconds, 57°C for 1 minute, 68°C for 2 minutes and a final extension of 68°C for 7 minutes. *TsetseEP* gives a product of a larger size when genomic DNA (indicative of a putative intron) was used as template (approximately 365 vs 315 bp. respectively) and was used to ensure genomic DNA was removed from experimental templates.

### Immunoblots

Immunoblotting using Hybond™-P polyvinylidene difluoride (PVDF) transfer membrane (Amersham Biosciences, Amersham, UK) was performed as previously described [36]. In brief, the primary antibodies used were either a 1∶20 dilution of anti-EP repeat mouse mAb TRBP1/247 [37]. The secondary (detecting) antibody was a 1∶50,000 dilution of horseradish peroxidase conjugated goat anti-mouse IgG/IgM (H+L) (Caltag Laboratories, South San Francisco, CA). Kodak Biomax MR film (Eastman Kodak Company, Rochester, NY) was used to detect chemiluminescence. After development of the autoluminograms, proteins were stained on the PVDF membrane with 0.2% (w/v) nigrosine in PBS. The exposed film was superimposed on the stained PVDF membrane to reveal the precise location of the immunoreactive protein bands in relationship to the entire protein profile and to ensure equivalent protein loading.

### Northern blots

Tsetse, which had fed twice, were injected on day 4 post emergence with 7 µg of gene-specific dsRNA (2 µl injection volume). Flies in the control group were injected with nuclease free water. Injected flies were fed again on day 5 and midguts dissected on day 7 were snap frozen in liquid nitrogen in pools of 5. The NorthernMax® formaldehyde-based system for Northern Blots (Ambion) was used. Total RNA (20 µg per lane) was loaded on a 1% formaldehyde-agarose gel. The Strip-EZ™ PCR probe synthesis and removal kit (Ambion) was used to synthesize single stranded DNA probes, which were labeled with [α^32^P] dATP (MP Biomedicals, Stretton Distributors, UK). Membranes were hybridized overnight at 42°C, given 2×5 minute low stringency washes and 2×15 minute high stringency washes before exposure to Kodak BioMax MR film.
